# Dental care and treatments provided under general anaesthesia in the Helsinki Public Dental Service

**DOI:** 10.1186/1472-6831-12-45

**Published:** 2012-10-27

**Authors:** Nora Savanheimo, Sari A Sundberg, Jorma I Virtanen, Miira M Vehkalahti

**Affiliations:** 1 Department of Oral Public Health, Institute of Dentistry, University of Helsinki, P.O. BOX 41, FI-00014, Helsinki, Finland; 2 Oral Health Care Department, City of Helsinki Health Centre, P.O. BOX 6000, 00099, City of Helsinki, Finland; 3 Department of Community Dentistry, Institute of Dentistry, University of Oulu, P.O. BOX 5281, FI-90014, Oulu, Finland; 4 Oulu University Hospital, FI-90220, Oulu, Finland

**Keywords:** Dental general anaesthesia, Public Dental Service, Indications, Comprehensive treatment, Preventive treatment, Immigration, Medically compromised patients

## Abstract

**Background:**

Dental general anaesthesia (DGA) is a very efficient treatment modality, but is considered only in the last resort because of the risks posed by general anaesthesia to patients’ overall health. Health services and their treatment policies regarding DGA vary from country to country. The aims of this work were to determine the reasons for DGA in the Helsinki Public Dental Service (PDS) and to assess the role of patient characteristics in the variation in reasons and in the treatments given with special focus on preventive care.

**Methods:**

The data covered all DGA patients treated in the PDS in Helsinki in 2010. The data were collected from patient documents and included personal background: age (<6, 6–12, 13–17, 18–68), gender, immigration, previous conscious sedation and previous DGA; medical background; reasons for DGA and treatments provided. Chi-square tests, Fisher’s exact test, and logistic regression modelling were employed in the statistical analyses.

**Results:**

The DGA patients (n=349) were aged 2.3 to 67.2 years. Immigrants predominated in the youngest age group (p<0.001) and medically compromised patients among the adults (p<0.001) relative to the other age groups. The main reason for DGA was extreme non-cooperation (65%) followed by dental fear (37%) and an excessive need for treatment (26%). In total, 3435 treatments were performed under DGA, 57% of which were restorations, 24% tooth extractions, 5% preventive measures, 5% radiography, 4% endodontics and the remaining 5% periodontics, surgical procedures and miscellaneous. The reasons for DGA and the treatments provided varied according to age, immigration, previous sedation and DGA and medical background. The logistic regression model showed that previous sedation (OR 2.3; 95%CI 1.3-4.1; p=0.005) and extreme non-cooperation (OR 1.7; 95%CI 0.9-3.2; p=0.103) were most indicative of preventive measures given.

**Conclusions:**

Extreme non-cooperation, dental fear and an excessive need for treatment were the main reasons for the use of comprehensive, conservative DGA in the Helsinki PDS. The reasons for the use of DGA and the treatments provided varied according to personal and medical background, and immigration status with no gender-differences. Preventive measures formed only a minor part of the dental care given under DGA.

## Background

Dental general anaesthesia (DGA) is a very efficient treatment modality, because it only takes a single appointment and requires little or no cooperation on the part of the patient. It is nevertheless considered only in the last resort, because general anaesthesia may pose risks for the patient’s overall health. General anaesthesia in early childhood has been reported to affect the child’s neurodevelopment, although contradictory findings have been reported [1,2].

The American Academy of Pediatric Dentistry (AAPD) has stated indications for DGA in children and adolescents as follows: (a) patients who cannot cooperate due to a lack of psychological or emotional maturity and/or mental, physical, or medical disability, (b) patients for whom local anaesthesia is ineffective because of acute infection, anatomical variations, or allergy, (c) patients who are extremely uncooperative, fearful, anxious, or uncommunicative, (d) patients who require significant surgical procedures or immediate, comprehensive oral/dental care and (e) patients for whom the use of DGA may protect the developing psyche and/or reduce the medical risk [3].

The AAPD and the Special Care Dentistry Association (SCDA) both emphasize that dentists should consider other techniques as alternatives to DGA and should use preventive care in order to find best treatment modality and achieve good results in the long term [3-5].

In Finland dental services are provided in both the public and the private sector, the entire population being entitled to Public Dental Service (PDS). Dental care for patients under 18 years of age is free of charge and nearly all children and adolescents receive PDS treatment. In Helsinki, DGA is provided by the PDS for ASA (American Society of Anesthesiologist) grade I-II patients, whereas ASA grade III-IV patients are referred to university hospitals. Conscious sedation is widely used when treating patients with difficulties in dental care, so that only those patients whose treatment would otherwise be very difficult are referred for out-patient DGA. At a consultation appointment preceding the treatment, a dentist specialised in DGA assesses, each patient individually in terms of the treatment options and needs, including proper instructions on oral self-care and dietary advice. Thus DGA is regarded as a comprehensive process, with preventive care included as one part.

In addition to the AAPD indications, the Helsinki PDS indications for the use of DGA with children and adolescents also recommend this approach for adolescents who are at risk of alienation from society due to dental problems and the need for extensive dental treatment and for adults with intellectual, physical, mental or medical disabilities that could be overcome this way.

The aims of this work were to determine the reasons for DGA in the Helsinki PDS and to assess the role of patient characteristics in the variation in reasons and in the treatments given with special focus on preventive care.

## Methods

### Subjects

The data covered all patients treated under DGA in the PDS in Helsinki, the capital of Finland, in 2010. The data were collected from patient referrals and other documents. Four patients were treated under DGA twice during the year and one patient three times. In these cases the multiple treatments were combined to represent one appointment. Complete documentation was available for every DGA patient.

The personal background data covered age to an accuracy of one month, gender, whether the individual was an immigrant or not, and the history of previous conscious sedation and/or DGA. Age was categorized in the analyses into four classes: <6, 6–12, 13–17 and 18–68 years, the first three describing eruptional stages in the dentition [6] and the fourth the age when patient started to pay medical fees. Immigrant status was defined in terms of nationality or native language.

### Medical background

The medical background data were extracted from the free format text contained in the patient documents and referrals. Medically compromised patients were recorded under five headings, allowing multiple records to be kept per patient: (a) intellectual disability, (b) behavioural disorders, (c) mental disorders, (d) physical limitations such as diseases of the nervous system or senses or musculoskeletal or connective tissue, and (e) other chronic medical conditions such as endocrine, nutritional, metabolic, infectious, circulatory, cardiac, digestive or respiratory system diseases. Allergies and surgical operations were not recorded. Medically compromised patients were categorized as having intellectual only (a+b+c), physical only (d+e), or both types of compromising conditions. For further analyses the patient’s medical background was dichotomized as having or not having any medically compromised conditions.

### Reasons for DGA

At the consultation appointment a DGA dentist assesses one or more reasons for treatment under DGA for each patient. The reasons were categorized for the present purpose as: extreme non-cooperation, extreme dental fear, an excessive need for treatment, avoidance of dental fear (for very young patients with no previous treatment experiences), large surgical procedures, a strong emetic reflex or ineffectiveness of local anaesthesia. Multiple reasons were allowed.

### Treatments under DGA

The data on the dental treatments performed under DGA were based on the patients’ documents. We recorded the number of restorations (including stainless steel crowns), extractions and endodontics (pulpotomies and root canal treatments) and surgical treatments (included surgical extractions, lingual and labial frenectomies, surgical removal of cystic lesions, buccal exostosis, odontoma and benign lesions in the oral soft tissue, tooth exposures, autotransplantations, placing of Bollard plates, excisions of hyperplastic tissue and cleaning of the incisive canal). Prophylaxis included professional tooth cleaning and/or topical application of chlorhexidine or fluoride. In addition, fissure sealants, periodontal therapy and radiographs taken during DGA were recorded separately. Prophylaxis and fissure sealants were combined under the heading of prevention. Miscellaneous treatments included alginate and precision impressions, adjustment of occlusal appliances, immediate complete dentures and Schwartz plates, repair of periodontal splints and other minor procedures.

### Ethical consideration

The ethics committee at the City of Helsinki Health Centre approved the study and granted full permission for it. Individuals were labelled with consecutive numbers for identification in the data analyses.

### Statistical analyses

The statistical analyses employed Chi-square tests, Fisher’s exact test, and logistic regression modelling.

## Results

### Description of patients

A total of 349 patients (185 male and 164 female) were treated under DGA in the Helsinki PDS in 2010. Their ages ranged from 2.3 to 67.2 years, with 31% under 6 years of age, 35% aged 6 to 12, 9% aged 13 to 17 and 25% aged 18 years or over (Table 1).

**Table 1:** Description of the patients (n=349) treated under dental general anaesthesia (DGA), by age group

**Characteristics of patients**	**Total**	**0-5 yr**	**6-12 yr**	**13-17 yr**	**18-68 yr**	***p***
	**n=349, %**	**n=108, %**	**n=123, %**	**n=30, %**	**n=88, %**	
*Gender*						
*Male*	53	55	52	43	56	*0.673*
*Female*	47	45	48	57	44	
*Immigrant*						
* Yes*	27	51	25	7	8	*<0.001*
* No*	73	49	75	93	92	
*Previous sedation*						
* Yes*	54	50	55	43	60	*0.318*
* No*	46	50	45	57	40	
*Previous DGA*						
* Yes*	20	1	14	37	48	*<0.001*
* No*	80	99	86	63	52	
*Medically compromised*						
* Yes*	39	12	24	63	86	*<0.001*
* No*	61	88	76	37	14	

Statistical evaluation by chi-square tests for differences by age group.

The patients’ characteristics are shown by age groups in Table 1. Immigrants predominated in the youngest age group, 51% compared with 25% in the 6-12-year-olds and 7-8% in the older groups (p<0.001). Of all the DGA patients 54% had previously received conscious sedation for dental care, with no age difference, whereas previous DGA was more frequent among the older patients (p<0.001).

Medically compromised patients predominated among the adults, 86%, whereas the vast majority of the 0-5-year-old (88%) and 6-12-year-old (76%) DGA-patients (p<0.001) had no medically compromising conditions. The mean proportion of all the DGA patients having one or more medically compromising conditions was 39% consisting of 14% intellectual conditions, 10% physical, and of 15% both. All three categories became more frequent the older the patients were (p<0.001).

### Reasons for DGA

The patient documentation revealed an average of 1.5 reasons for DGA per patient, 54% having one reason, 40% two and 6% three. Among the adult patients 73% had one reason for DGA, as compared with 48% and 49% in the two youngest age groups (p=0.002).

The reasons for DGA by age of the patients are shown in Table 2. The main reason was extreme non-cooperation (65%) followed by extreme dental fear (37%) and an excessive need for treatment (26%). This rank order was the same for all age groups except the youngest one, where avoidance of dental fear was the reason in 27% of cases and for 18–68 year-olds, where a strong emetic reflex was the reason for 15% of cases. Age was a powerful determinant for most of the reasons, but no gender-differences were found.

**Table 2:** Reasons for dental general anaesthesia (DGA), by age group

**Reasons for DGA**	**Total**	**0-5 yr**	**6-12 yr**	**13-17 yr**	**18-68 yr**	***p***
	**n=349, %**	**n=108, %**	**n=123, %**	**n=30, %**	**n=88, %**	
*Extreme non-cooperation*	65	75	65	60	55	*0.025*
*Extreme dental fear*	37	21	43	60	40	*<0.001*
*Excessive need for treatment*	26	35	27	33	11	*0.002*
*Avoidance of dental fear*	10	27	5	0	0	*<0.001* F
*Large surgical procedures*	7	2	14	7	5	*0.003* F
*Strong emetic reflex*	6	1	5	0	15	*<0.001* F
*Ineffectiveness of local anesthesia*	1	0	1	3	3	*0.100* F

For each patient, one or more reasons for referring to DGA were documented.

Statistical evaluation by chi-square tests for differences by age group. F=Fisher’s exact test (F) applied.

The three most common reasons for treatment under DGA are shown in relation to the patients’ characteristics in Table 3. An immigrant background was more often indicative of an excessive need for treatment than a non-immigrant one (37% vs. 22%; p=0.005) and was less often indicative of a dental fear (27% vs. 41%; p=0.023). Those with previous experience of sedation more often showed extreme non-cooperation and dental fear than their non-sedated counterparts but less often had an excessive need for treatment. Likewise an excessive need for treatment was less frequent for those with previous DGA and/or medically compromising condition, than for those with no previous DGA (10% vs. 30%; p<0.001) or with no medical problems (18% vs. 31%; p=0.007).

**Table 3:** The most common reasons for dental general anaesthesia (DGA), by patient characteristics

**Reasons for DGA**	**Immigrant**		**Previous sedation**		**Previous DGA**		**Medically compromised**	
	**Yes**	**No**	**Yes**	**No**	**Yes**	**No**	**Yes**	**No**
	**n=95, %**	**n=254, %**	**n=188, %**	**n=161, %**	**n=71, %**	**n=278, %**	**n=137, %**	**n=212, %**
*Extreme non-*	71	63	79	49	68	64	62	67
*cooperation*	*p=0.189*		*p<0.001*		*p=0.612*		*p=0.345*	
*Extreme dental*	27	41	43	30	31	38	33	40
*fear*	*p=0.023*		*p=0.011*		*p=0.242*		*p=0.200*	
*Excessive need*	37	22	18	35	10	30	18	31
*for treatment*	*p=0.005*		*p<0.001*		*p<0.001*		*p=0.007*	

Statistical evaluation by chi-square tests for differences according to patient characteristics.

### Treatments under DGA

A total of 3435 treatments were performed under DGA, of which 57% were restorations, 24% tooth extractions, 5% preventive measures, 5% radiography and 4% endodontics, the remaining 5% being periodontics, surgical procedures and miscellaneous. The mean number of treatments per patient was 9.8 (SD 5.0), ranging from 8.8 (SD 3.9) for 6-12-year-olds to 11.0 (SD 5.6) for 13-17-year-olds. Most of the treatments were restorations (5.6, SD 3.6) and extractions (2.3, SD 3.3).

**Figure 1:**
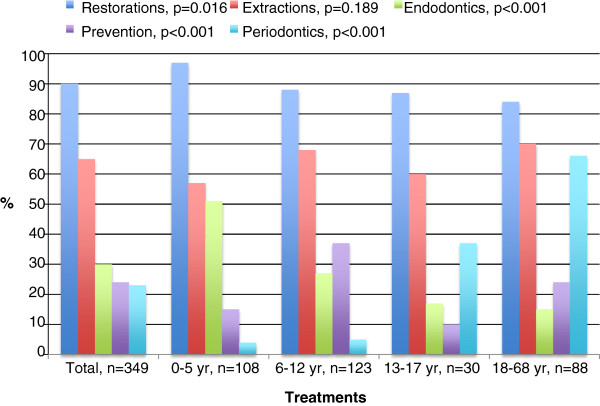
**Percentages (%) of dental general anaesthesia patients (n=349) receiving various treatments, by age group.** P-values refer to differences between the age groups.

The percentages of patients who received each type of treatment are presented by age in Figure 1. The youngest age group dominated those receiving filling therapy (97% vs. 84-88%; p=0.016) and endodontic treatment (51% vs. 15-27%; p<0.001), the 6-12-year-olds those receiving preventive treatment (37% vs. 10-24%; p<0.001) and the adults those receiving periodontic treatment (66% vs. 4-37%; p<0.001). Altogether 26% of the patients had radiographs taken under DGA.

The treatments received under DGA are shown by patient characteristics in Table 4. Being an immigrant pointed to filling therapy and endodontics more often than being a non-immigrant but less to periodontics, while those with previous sedation more often received filling therapy, preventive measures or periodontics than their non-sedated counterparts and those with previous DGA less often received filling therapy or endodontic treatment but more preventive measures or periodontics. The medically compromised DGA patients received filling therapy and endodontic treatment less often than did those without medical problems, but periodontic treatment more often.

**Table 4:** Treatments provided for dental general anaesthesia (DGA) patients (n=349) by patient characteristics

**Treatments received**	**Total**	**Immigrant**		**Previous sedation**		**Previous DGA**		**Medically compromised**	
	**n=349, %**	**Yes**	**No**	**Yes**	**No**	**Yes**	**No**	**Yes**	**No**
		**n=95, %**	**n=254, %**	**n=188, %**	**n=161, %**	**n=71, %**	**n=278, %**	**n=137, %**	**n=212, %**
*Filling*	90	100	86	93	86	82	92	85	93
*therapy*		*p<0.001*		*p=0.024*		*p=0.013*		*p=0.013*	
*Tooth*	65	66	64	65	64	55	67	66	64
*extraction*		*p=0.709*		*p=0.777*		*p=0.052*		*p=0.768*	
*Endodontics*	30	47	24	29	32	14	35	23	35
		*p<0.001*		*p=0.624*		*p<0.001*		*p=0.011*	
*Prevention*	24	31	22	32	16	34	22	26	24
		*p=0.101*		*p<0.001*		*p=0.038*		*p=0.677*	
*Periodontics*	23	11	27	28	17	51	15	45	8
		*p<0.001*		*p=0.015*		*p<0.001*		*p<0.001*	

Statistical evaluation by chi-square tests for differences according to patient characteristics.

The treatments received by the patients are shown by reasons for DGA in Table 5. Those with extreme non-cooperation, extreme dental fear or an excessive need for treatment received filling therapy more often than those without these properties, while endodontic and preventive treatments were more common for those with extreme non-cooperation and tooth extraction and periodontics for those with an excessive need for treatment.

**Table 5:** Treatments provided for dental general anaesthesia (DGA) patients (n=349) by reasons for DGA

**Treatments received**	**Total**	**Extreme non-cooperation**		**Extreme dental fear**		**Excessive need for treatment**	
	**n=349, %**	**Yes**	**No**	**Yes**	**No**	**Yes**	**No**
		**n=227, %**	**n=122, %**	**n=129, %**	**n=220, %**	**n=91, %**	**n=258, %**
*Filling therapy*	90	94	81	95	86	97	87
		*p<0.001*		*p=0.008*		*p=0.010*	
*Tooth extraction*	65	66	62	70	62	74	62
		*p=0.480*		*p=0.134*		*p=0.039*	
*Endodontics*	30	34	24	26	33	34	29
		*p=0.049*		*p=0.212*		*p=0.373*	
*Prevention*	24	30	15	19	28	23	25
		*p=0.002*		*p=0.055*		*p=0.741*	
*Periodontics*	23	24	20	21	24	14	26
		*p=0.483*		*p=0.560*		*p=0.027*	

Statistical evaluation by chi-square tests for differences according to reasons for dental GA.

The roles of patient characteristics and the reasons for DGA with regard to the provision of preventive treatment during the DGA session are shown in Table 6. Previous sedation was more frequently indicative of preventive treatment (OR=2.3; 95%Cl 1.3-4.1; p=0.005) and tooth extraction less frequently (OR=0.9; 95%Cl 0.8-0.98; p=0.022). Extreme non-cooperation tended to be indicative of prevention, whereas extreme dental fear tended to result in less preventive measures.

**Table 6:** Factors explaining the provision of preventive treatment under dental general anaesthesia in logistic regression modelling

**Parameter**	**Estimate**	**SE**	**OR**	**95%CI**	***p***
Being immigrant: 0=No, 1=Yes	0.469	0.31	1.6	0.9, 2.9	*0.128*
Previous sedation: 0=No, 1=Yes	0.830	0.30	2.3	1.3, 4.1	*0.005*
Receiving tooth extraction: 0=No, 1=Yes	−0.139	0.06	0.9	0.8, 0.98	*0.022*
Extreme non-cooperation: 0=No, 1=Yes	0.532	0.33	1.7	0.9, 3.2	*0.103*
Extreme dental fear: 0=No, 1=Yes	−0.473	0.30	0.6	0.3, 1.1	*0.110*
Excessive need for treatment: 0=No, 1=Yes	0.353	0.33	1.4	0.7, 2.7	*0.283*
Constant term	−1.975	0.43			

Deviance=357.2; df=340.

In the fitted model, age and gender were controlled for.

## Discussion

Extreme non-cooperation and extreme dental fear were the most important factors leading to DGA, and should, therefore be taken into account and prevented early on in order to reduce the need for DGA. The present findings based on a unique body of PDS data covering all age groups support earlier observations that dental fear, non-cooperation, compromising medical conditions and the need for extensive dental treatment are the most common reasons for out-patient DGA [7-12]. From the parents’ point of view, dental fear and repeated unpleasant experiences during dental treatment can lead to a utilisation for DGA even in the case of healthy children [13].

One of the commonest among the many reasons lying behind non-cooperation in children is dental fear [14]. It has been reported in Finland that 21-36% of children are quite afraid or very afraid of something connected with dental treatment [15] and that 5-19% of adults are very afraid of visiting a dentist [16], while correspondingly one fourth of adults in England reported that they definitely always feel anxious about going to a dentist [17]. Non-cooperative and fearful patients need more time and effort on the part of the dental team. Many of the present patients had previously received dental treatment under conscious sedation, indicating that dentists had tried to treat their dental fear prior to resorting to DGA. As DGA does not diminish dental fear, as reported in children [18], dental fear needs to be dealt with after DGA. Patients in the Helsinki PDS are scheduled for a post-DGA appointment, which simulates a normal dental situation, with the intention of guiding the patient back to normal dental care. In addition, proper oral self-care instructions and dietary advice are provided over again.

DGA in the Helsinki PDS context was a comprehensive, conservative process characterized by a predominance of filling therapy, endodontics and periodontics. Comprehensive DGA has earlier been reported in many European countries [8,9,11,19-21], North America [7,22,23], the Middle East [24,25], Asia [26,27] and New Zealand [28]. Contradictory findings have recently been reported from Australia and England, where DGA is used primarily for extractions in both children and adults [29-31], although a move towards comprehensive DGA care has also been made in the United Kingdom since the publication of the Royal College of Surgeon’s guidelines for the use of GA in paediatric dentistry in 2008 [32].

Nearly one fourth of the patients received preventive treatment under DGA, but the overall proportion of this, 5% was relatively small. One explanation may be that tooth extractions performed in this way do not allow prophylaxis at the same time. Fissure sealants and prophylaxis have been reported earlier as part of a comprehensive dental care regimen performed under DGA on children [7,10,11], but among our patients nearly one fourth of the adults received preventive treatment, too.

Half of our adult patients had previously been treated under DGA, and 86% of the adults had a medically compromising condition, indicating that they belonged to a group whose dental care must necessarily be performed under DGA. Dougherty [33] states that the decision as to which treatment modality is in the best interest of a patient with special needs should be made individually, but there is little evidence for what might be the optimal frequency of treatment episodes under DGA.

Our findings were based on patient documents and referrals, which normally include no information about socioeconomic status. Immigration status is noted, however, and may be used as an indication of cultural differences which may affect oral health and related behaviour. The fact that immigrant children were over-represented among our young DGA patients is in line with an earlier report from Denmark [12]. By contrast, an Australian report states that indigenous children have a higher risk of receiving DGA [19]. To reduce inequalities in the use of health services, the Helsinki PDS has initiated multiprofessional collaboration in a programme that provides information on dental care and prevention for immigrant families and education in cultural disparities for PDS personnel.

City of Helsinki statistics show that around 160 000 out of a total of almost 600 000 Helsinki residents were treated in the PDS in 2010 and that 349 of these were DGA patients, indicating that DGA is used as a last resort and only when certain strict criteria have been fulfilled. Our comprehensive data on DGA treatments provided during one-year are representative of the situation in the Helsinki PDS and may be generalized for the whole country, since Helsinki residents make up over 10% of Finland’s population. The data were based on the patients’ dental documents, the compiling of which is governed by strict rules in Finland.

## Conclusions

Extreme non-cooperation, dental fear and an excessive need for treatment were the main reasons for the use of comprehensive, conservative DGA in the Helsinki PDS. The reasons for the use of DGA and the treatments provided varied according to personal and medical background, and immigration status with no gender-differences. Preventive measures were more frequently performed on patients with previous experience of conscious sedation or extreme non-cooperation, but these measures formed only a minor part of the dental care given under DGA.

## Competing interests

The author(s) declare that they have no competing interests.

## Authors’ contributions

NS: designed the study, collected data and wrote the manuscript, SAS: designed the study and collected data, JIV: designed the study and wrote the manuscript, MMV: designed the study, performed statistical analyses and wrote the manuscript. All authors read and approved the final manuscript.

## Pre-publication history

The pre-publication history for this paper can be accessed here:

http://www.biomedcentral.com/1472-6831/12/45/prepub

## References

[B1] Sun L Early childhood general anaesthesia exposure and neurocognitive development Br J Anaesth 2010 105 Suppl 1 i61 i68 21148656 10.1093/bja/aeq302 PMC3000523

[B2] Stratmann G Review article: Neurotoxicity of anesthetic drugs in the developing brain Anesth Analg 2011 113 1170 1179 10.1213/ANE.0b013e318232066c 21965351

[B3] The American Academy of Pediatric Dentistry Guideline on behavior guidance for the pediatric dental patient 2011 http://www.aapd.org.

[B4] American Academy on Pediatric Dentistry Council on Clinical Affairs Guideline on management of dental patients with special health care needs Pediatr Dent 2008–2009 30 7 Suppl 107 111 19216407

[B5] Glassman P Caputo A Dougherty N Lyons R Messieha Z Miller C Peltier B Romer M Special Care Dentistry Association consensus statement on sedation, anesthesia and alternative techniques for people with special needs Spec Care Dentist 2009 29 2 8 10.1111/j.1754-4505.2008.00055.x 19152561

[B6] Haavikko K The formation and the alveolar and clinical eruption of the permanent teeth. An orthopantomographic study 1970 University of Helsinki: PhD Thesis 4917152

[B7] Legault JV Diner MH Auger R Dental treatment of children in a general anaesthesia clinic: review of 300 cases J Can Dent Assoc (Tor) 1972 38 221 224 4260583

[B8] Grytten J Holst D Dyrberg L Fæhn O Some characteristics of patients given dental treatment under general anesthesia Acta Odontol Scand 1989 47 1 5 10.3109/00016358909004793 2718750

[B9] Tarján I Mikecz G Dénes J General anaesthesia of out-patients in pedodontics J Int Assoc Dent Child 1990 20 59 61 2151817

[B10] Nunn JH Davidson G Gordon PH Storrs J A retrospective review of a service to provide comprehensive dental care under general anesthesia Spec Care Dentist 1995 15 97 101 10.1111/j.1754-4505.1995.tb00489.x 8619175

[B11] Vinckier F Gizani S Declerck D Comprehensive dental care for children with rampant caries under general anaesthesia Int J Paediatr Dent 2001 11 25 32 10.1046/j.1365-263x.2001.00204.x 11309869

[B12] Haubek D Fuglsang M Poulsen S Rølling I Dental treatment of children referred to general anaesthesia – association with country of origin and medical status Int J Paediatr Dent 2006 16 239 246 10.1111/j.1365-263X.2006.00737.x 16759320

[B13] Savanheimo N Vehkalahti MM Pihakari A Numminen M Reasons for and parental satisfaction with children’s dental care under general anaesthesia Int J Paediatr Dent 2005 15 448 454 10.1111/j.1365-263X.2005.00681.x 16238655

[B14] Baier K Milgrom P Russell S Mancl L Yoshida T Children’s fear and behavior in private pediatric dentistry practises Pediatr Dent 2004 26 316 321 15344624

[B15] Rantavuori K Aspects and determinants of children’s dental fear 2008 University of Oulu, Faculty of Medicine, Institute of Dentistry: PhD thesis

[B16] Lahti S Vehkalahti MM Nordblad A Hausen H Dental fear among population aged 30 and older in Finland Acta Odontol Scand 2007 65 97 102 10.1080/00016350601058085 17453427

[B17] Kelly M Steele J Nuttall N Bradnock G Morris J Nunn J Pine C Pitts N Treasure E White D Adult Dental Health Survey. Oral Health in the United Kingdom 1998. Office for National Statistics 2000 London: The Stationary Office

[B18] Klaassen MA Veerkamp JSJ Hoogstraten J Young children’s Oral Health-Related Quality of Life and dental fear after treatment under general anaesthesia: a randomized controlled trial Eur J Oral Sci 2009 117 273 278 10.1111/j.1600-0722.2009.00627.x 19583755

[B19] Vermeulen M Vinckier F Vandenbroucke J Dental general anesthesia: clinical characteristics of 933 patients ASDC J Dent Child 1991 58 27 30 1827804

[B20] Pohl Y Filippi A Geiger G Kirschner H Boll M Dental treatment of handicapped patients using endotracheal anesthesia Anesth Prog 1996 43 20 23 10323121 PMC2153453

[B21] Harrison MG Roberts GJ Comprehensive dental treatment of healthy and chronically sick children under intubation general anaesthesia during a 5-year period Br Dent J 1998 184 503 506 10.1038/sj.bdj.4809675 9642869

[B22] Enger DJ Mourino AP A survey of 200 pediatric dental general anesthesia cases ASDC J Dent Child 1985 52 36 41 3856585

[B23] Loyola-Rodriguez JP Zavala-Alonso V Gonzalez-Alvarez CL Juarez-Lopez LA Patiño-Marin N Gonzalez CD Dental treatment under general anesthesia in healthy and medically compromised/developmentally disabled children: a comparative study J Clin Pediatr Dent 2009 34 177 182 20297713 10.17796/jcpd.34.2.u665328k4g467pg2

[B24] Ibricevic H Al-Jame Q Honkala S Pediatric dental procedures under general anesthesia at the Amiri hospital in Kuwait J Clin Pediatr Dent 2001 25 337 342 11497018 10.17796/jcpd.25.4.fl062x558qtt4v69

[B25] Jamjoom MM Al-Malik MI Holt RD El-Nassry A Dental treatment under general anaesthesia at a hospital in Jeddah, Saudi Arabia Int J Paediatr Dent 2001 11 110 116 11310133 10.1046/j.1365-263x.2001.00252.x

[B26] Kwok-Tung L King NM Retrospective audit of caries management techniques for children under general anesthesia over an 18-year period J Clin Pediatr Dent 2006 31 58 62 17091661 10.17796/jcpd.31.1.956272nw2864021p

[B27] Lee PY Chou MY Chen YL Chen LP Wang CJ Huang WH Comprehensive dental treatment under general anesthesia in healthy and disabled children Chang Gung Med J 2009 32 636 642 20035643

[B28] Drummond BK Davidson LE Williams SM Moffat SM Ayers KM Outcomes two, three and four years after comprehensive care under general anaesthesia N Z Dent J 2004 100 32 37 15346870

[B29] Jamieson LM Roberts-Thomson KF Dental general anaesthetic trends among Australian children BMC Oral Health 2006 6 16 17184552 10.1186/1472-6831-6-16 PMC1770909

[B30] Jamieson LM Roberts-Thomson KF Dental general anaesthetic receipt among Australians aged 15+ years, 1998–1999 to 2004–2005 BMC Oral Health 2008 8 10 18402707 10.1186/1472-6831-8-10 PMC2329614

[B31] Moles DR Ashley P Hospital admissions for dental care in children: England 1997–2006 Br Dent J 2009 206 E14 10.1038/sj.bdj.2009.254 19330014

[B32] Davies C Harrison M Roberts G UK national clinical guidelines in paediatric dentistry: guideline for the use of general anaesthesia (GA) in paediatric dentistry 2008 London: Royal College of Surgeons of England

[B33] Dougherty N The dental patient with special needs: a review of indications for treatment under general anesthesia Spec Care Dentist 2009 29 17 20 10.1111/j.1754-4505.2008.00057.x 19152563

